# Evaluation of shoot-growth variation in diverse carrot (*Daucus carota* L.) germplasm for genetic improvement of stand establishment

**DOI:** 10.3389/fpls.2024.1342512

**Published:** 2024-04-19

**Authors:** Jenyne Loarca, Michael Liou, Julie C. Dawson, Philipp W. Simon

**Affiliations:** ^1^ Vegetable Crops Research Unit, United States Department of Agriculture, Madison, WI, United States; ^2^ Department of Plant and Agroecosystem Sciences, University of Wisconsin–Madison, Madison, WI, United States; ^3^ Department of Statistics, University of Wisconsin–Madison, Madison, WI, United States

**Keywords:** plant genetic resources, weed competition, seedling emergence, diverse germplasm, crop resilience, diversity panel, crop wild relatives, crop ontology

## Abstract

Carrot (*Daucus carota* L.) is a high value, nutritious, and colorful crop, but delivering carrots from seed to table can be a struggle for carrot growers. Weed competitive ability is a critical trait for crop success that carrot and its apiaceous relatives often lack owing to their characteristic slow shoot growth and erratic seedling emergence, even among genetically uniform lines. This study is the first field-based, multi-year experiment to evaluate shoot-growth trait variation over a 100-day growing season in a carrot diversity panel (N=695) that includes genetically diverse carrot accessions from the United States Department of Agriculture National Plant Germplasm System. We report phenotypic variability for shoot-growth characteristics, the first broad-sense heritability estimates for seedling emergence (0.68 < H^2^ < 0.80) and early-season canopy coverage ( 0.61 < H^2^ < 0.65), and consistent broad-sense heritability for late-season canopy height (0.76 < H^2^ < 0.82), indicating quantitative inheritance and potential for improvement through plant breeding. Strong correlation between emergence and canopy coverage (0.62 < r < 0.72) suggests that improvement of seedling emergence has great potential to increase yield and weed competitive ability. Accessions with high emergence and vigorous canopy growth are of immediate use to breeders targeting stand establishment, weed-tolerance, or weed-suppressant carrots, which is of particular advantage to the organic carrot production sector, reducing the costs and labor associated with herbicide application and weeding. We developed a standardized vocabulary and protocol to describe shoot-growth and facilitate collaboration and communication across carrot research groups. Our study facilitates identification and utilization of carrot genetic resources, conservation of agrobiodiversity, and development of breeding stocks for weed-competitive ability, with the long-term goal of delivering improved carrot cultivars to breeders, growers, and consumers. Accession selection can be further optimized for efficient breeding by combining shoot growth data with phenological data in this study’s companion paper to identify ideotypes based on global market needs.

## Introduction

Carrot (*Daucus carota* L.) is a widely grown vegetable crop that provides consumers with an affordable rich supply of nutrients (Drewnowski, 2013). Carrot is in the top nine most nutrient rich and cost-accessible vegetables, it is an exceptional source of beta-carotene, which functions as a provitamin A carotenoid, and offers appreciable quantities of B vitamins (thiamin, riboflavin, and niacin) compared with other commonly consumed vegetables, and is a good source of fiber (Arscott and Tanumihardjo, 2010; Drewnowski, 2010; Suchánková et al., 2015). Carrot breeding programs have prioritized breeding for traits that improve taproot quality and yield, such as flavor, color, texture, disease resistance, and pest resistance (Rubatzky et al., 1999). Excellent root quality has been a driver of carrot’s wide commercial acceptance and high frequency of use (Drewnowski, 2013). However, while foliar and root diseases have received much attention by carrot researchers, little attention has been paid to the above-ground vegetative growth. Shoot vigor traits have recently become a priority for carrot growers, particularly those growers in the organic sector, who grow crops with limited weed control options. Apiaceous crops like carrot are characterized by erratic seed germination, slow emergence, slow growth, and delayed canopy closure (Rubatzky et al., 1999; Colquhoun et al., 2017). Shoot architecture and shoot biomass affect light acquisition, and consequently, slow growth limits season-long weed-competitive ability, plant growth, development, and crop productivity. Moreover, weed interference causes misshapen roots, thereby decreasing root quality and reducing root yield.

Few published studies have evaluated genetic improvement of crop stand establishment, as it has historically been managed with horticultural practices (Maynard et al., 2006). While these practices are often costly, laborious, and time-consuming, they have been the preferred management method because of their potential to provide more immediate relief than plant breeding, which is a long term strategy that requires significant investment in resources and time. However, once achieved, plant breeding solutions have the potential to save significant costs in time, and labor. To date, most studies on carrot stand establishment have attempted to address the problem with weed management strategies or by developing treatments that increase carrot germination rate and uniformity.

In general, practices that improve crop stand establishment include irrigation methods in maize (El-Sanatawy et al., 2021), planting date by variety interaction in alfalfa (Beveridge and Wilsie, 1959), and planting depth in cereals (Hadjichristodoulou et al., 1977). Other relevant practices include intercropping, weed removal, and overplanting to compensate for poor germination or emergence. Environmental factors such as temperature, moisture, pathogens, pests, and soil health are examples of important factors that affect crop establishment (Grassbaugh and Bennett, 1998). Soil composition, soil crusting, soil water-holding capacity, and soil heterogeneity can create impedances to carrot seedling emergence if not properly managed (Hegarty, 1979). As such, bed preparation and moisture availability are critical to stand establishment. Soils with high water-holding capacity, such as heavy clay soils, are prone to crusting, which creates a hard impenetrable physical barrier through which successfully growing seedlings are unable to emerge. Sandy soils can still form a crust if not sufficiently tilled. Although sandy soil is well-draining, it has a low water-holding capacity; however, this can be mitigated with sufficient watering.

Moisture availability is also a critical factor in post-germination and pre-emergence carrot growth in the field (Finch-Savage and Pill, 1990; Finch-Savage et al., 1998), which is why seed priming is a prevalent area of research in carrot. In carrot, immature embryo dormancy partially contributes to asynchronous germination, with mature seed germinating 3.7 days earlier than immature seed (Brocklehurst and Dearman, 1980). Seed priming functions by imbibing the seed with water, which promotes the activation stage of germination, then drying the seed for later planting. This method allows less-developed seed, which may take more time to reach activation, to achieve this stage; the process synchronizes the differently matured seed. Seed priming and other seed treatments are often used to improve the rate and percentage of germination, which leads to increased synchrony and speed of seedling emergence and crop establishment (Rubatzky et al., 1999; Prohens and Nuez, 2008). Other seed treatments include seed pelleting, film coating, and fungicide treatments (Maynard et al., 2006). Even with added cost, growers see enough benefit to justify seed treatment in many cases. The positive impact of seed treatments on crop germination have been researched in many crops, such as barley (Abdulrahmani et al., 2007), corn (El-Sanatawy et al., 2021), soybean (Arif et al., 2008), rice (Farooq et al., 2006; Ella et al., 2011), cowpea (Eskandari and Kazemi, 2011), table beet (Khan et al., 1992), and lettuce (Seale and Cantliffe, 1986). Osmotic priming has been used effectively in Apiaceous crops such as celery (Salter and Darby, 1976), parsnip (Gray et al., 1984), and carrot (Finch-Savage and McQuistan, 1988; Sanders et al., 1990; Bennett et al., 1992). Seed treatments that improve emergence and yield in carrot field studies include osmotic priming (Szafirowska et al., 1981), hormonal priming (Lada et al., 2004), and seed priming with salicylic acid (Mahmood-ur-Rehman et al., 2020). Recent research has focused on carrot seed quality screening (Marchi and Cicero, 2017) and seed treatment to improve germination (Aazami and Zahedi, 2018; Sowmeya et al., 2018; Mahmood-ur-Rehman et al., 2020; Guragain et al., 2021; Muhie et al., 2021; Muhie et al., 2024). These treatments make significant, but fractional, improvements to total carrot germination and uniform carrot germination. And while high germination rate is a necessary condition of high emergence, it does not assure high emergence.

Studies on horticultural control of weeds among carrots also outnumber studies on genetic control of carrot weed competitive ability. Weed management is a critical and proactive approach to mitigate carrot crop losses to competition, as carrot is the most sensitive (among 26 crops studied) to weed interference (Van Heemst, 1985). The first six weeks of the carrot growth cycle is known as the critical weed-free period, when slow-growing carrots are most susceptible to competition from weeds (Swanton et al., 2010). Chemical control of weeds is a common strategy in conventional carrot growing operations, but few herbicides are designated specifically for carrot. Among many herbicides (Colquhoun et al., 2019), one such example, linuron, is used for broadleaf weed control for carrot 3 - 6 weeks post-emergence. However, linuron requires that carrot shoots to achieve a threshold height of 7.6 cm before application, which is typically achieved around the fifth or sixth week after planting, which is fairly late into the critical weed-free period (Bellinder et al., 1997). In addition, lack of herbicide rotation has had an ecological impact due to the evolution of linuron-resistant pigweed populations (Colquhoun et al., 2017). Linuron is also not a management option in organic carrot growing operations (Colquhoun et al., 2017), which represent 14% of U.S. carrot production (USDA Economic Research Service (ERS), 2023), so machine- and/or hand-weeding are management strategies for organic growers. Without weeding, carrot yield losses can range from 38% - 87% (Colquhoun et al., 2017). Within-row weeding also removes late-emerging carrot seedlings that act as weed-like competitors to earlier emerged seedlings. However, hand-weeding is laborious, costly, time-intensive, and disruptive to established seedlings. Moreover, despite continuous hand-weeding, growers may still suffer an average yield loss of 15% (Colquhoun et al., 2017). Recent studies maintain focus on weed management strategies to reduce competition with carrot shoots (De Boer et al., 2019; Miao et al., 2019; Colquhoun, 2020; Ying et al., 2021; Mou et al., 2023).

Identifying genetically-controlled carrot traits that increase weed competitive ability would enable breeders to deliver carrot cultivars that help solve the issue of weed-related yield losses for growers. For a plant breeding approach to be effective, the attributes must be heritable, and so understanding the heritability of carrot growth attributes that confer weed tolerance/suppression, such as vigorous growth, uniform emergence, and early canopy closure is essential (Colquhoun et al., 2017). There is further interest in dissecting correlation among weed-competitive traits and carrot yield, which are poorly understood given the limited number of studies on genetic architecture of carrot shoot growth. One of the earliest studies on carrot shoot and root characteristics found that early emerging seedlings tended to have less root size variation at harvest (Mann & MacGillivray, 1949) and that seed weight in turn correlates positively with improved emergence and high early root yield (Austin and Longden, 1967). Consistently, other studies found that variation in embryo size, spread of emergence, seedling size and weight at emergence all influenced taproot weight variability at harvest, but seed weight and size did not (Gray and Steckel, 1983a; Gray et al., 1986, 1983b; Salter et al., 1981). Variability in embryo length is also a reliable early indicator of root crop uniformity (Dowker, 1978). Dowker (1978) acknowledged that embryo length heritability of seedling traits had not been calculated. In a study of one cultivar, Gray (1984) misinterpreting Dowker, claimed that genetic factors of embryo length were not important and that variation in embryo length is not influenced by the genetic constitution of the seed. It is correct that non-genetic factors influence embryo length variation, and include umbel order and seed harvest date, both of which are controlled through seed-parent planting density and seed harvesting methods that eliminate underdeveloped seed (Gray and Steckel, 1985). Non-genetic sources of embryo length variation include umbel order and seed harvest date, both of which are controlled through seed-parent planting density and seed harvesting methods that eliminate underdeveloped seed (Gray and Steckel, 1985). A recent study found that 0.6% of variation in emergence is explained by varietal identity, and 70% by environmental factors, concluding that environmental conditions are more important than genetic background or intrinsic seed quality (Hundertmark-Bertaud et al., 2019). However, because Hundertmark-Bertaud only used five, genetically similar, F1 varieties of the same market class, there was likely insufficient genetic variation present to substantiate their claim that genetic factors are universally less important and environmental factors, or whether their conclusions were generalizable or not to other carrot populations, particularly those with higher genetic diversity.

There has been little research dedicated to unraveling the genetics of stand establishment traits in carrot, but a few recent studies provide strong supporting evidence that these traits have heritable variation. Carrot growers have long recognized large differences in canopy size among commercial cultivars (Simon et al., 2017). That study reported wide ranges for canopy size and canopy height in a diverse carrot germplasm collection that included purple, yellow, and red colored roots, open-pollinated varieties, segregating filial generations (F_2_-F_5_ populations), and inbred lines from the United States Department of Agriculture - Agricultural Research Service (USDA-ARS) Vegetable Crops Research Unit (VCRU) (Colquhoun et al., 2017), whose study of nine commercial carrot varieties were evaluated for weed-suppressive traits in the early-mid season (Colquhoun et al., 2017). Their study observed significant differences in emergence rate, canopy development (ground cover), and weed tolerance. Genetic variation for late-season carrot shoot growth and shoot architecture has also been documented by Turner et al. (2018a), who developed an imaging pipeline that extracts size and shape traits for carrot shoots, including shoot morphology, petiole number, petiole length, petiole width, and biomass. This pipeline was then used to phenotype a F2 mapping population (N = 316) that segregated for various shoot traits, leading to the identification of quantitative trait loci (QTL) for shoot characteristics on chromosomes 1, 2, and 7, suggesting genetic control of shoot growth (Turner et al., 2018b). Canopy height variation at harvest was documented by Luby et al. (2016), who studied a panel of commercially available U.S. carrot varieties (N = 140) and found broad-sense heritability to be 0.82, suggesting that genetic factors contribute to end-of-season top height variation (Luby et al., 2016). An in-depth literature review of crop stand establishment studies can be accessed for further information (Loarca, 2021).

An extensive review of global *Daucus* germplasm collections is provided by Allender (2019). Globally, *ex situ Daucus* germplasm collections are extensive, with more than 13,400 accessions conserved (not yet accounting for duplicated material) across 62 institutions. Global *Daucus* genetic resources have been collected from over 75 countries, though sampling depth is low from Africa and South America (Allender, 2019; Mezghani et al., 2019). According to the Genesys database, the USDA carrot germplasm collection (1381 accessions) is among the largest *Daucus* collections, with about 695 accessions classified as cultivated and the rest wild relatives. Other international carrot germplasm collections such as the German genebank at the Leibniz Institute of Plant Genetics and Crop Plant Research (493 *Daucus* accessions), the Plant Breeding and Acclimatization Institute in Poland (629 *Daucus* accessions), and the UK Vegetable Genebank (1457 *Daucus* accessions), which was designated at the world base for carrot germplasm by the International Board for Plant Genetic Resources (IBPGR, now Bioversity). Other notable *Daucus* collections that are not cataloged by Genesys are maintained by the Vavilov Institute in Russia (3102 accessions), the Institute of Vegetables and Flowers at Chinese Academy of Agricultural Sciences (~400 accessions), the National Bureau of Plant Genetic Resources in India, and a national network ‘Carrot and other *Daucus* genetic resources’ (3131 accessions) in France. It was not clear from Allender’s review what proportions of these collections are cultivated carrot or carrot crop wild relatives (CWR).

This study is the first and largest (N = 695 accessions) multi-year field evaluation of agronomically important shoot growth traits spanning an entire field season, from germination to emergence to harvest, in diverse carrot germplasm that includes landraces. The carrot accessions (also known as plant introductions) in this study are maintained by the United States Department of Agriculture National Plant Germplasm System (USDA-NPGS), which is a major source of useful plant genetic resources (PGR) for breeding programs, and yet one of the major barriers to using PGR is accession evaluation (Byrne et al., 2018). All or parts of this global USDA germplasm collection have previously been evaluated in studies on canopy vigor (Loarca et al., 2024), core collection curation (Corak et al., 2019), demographic history of carrot domestication and breeding (Coe et al., 2023), genetic structure, phyologeny, and carotenoid presence (Ellison et al., 2018), taproot shape (Brainard et al., 2021), plant growth traits (Acosta-Motos et al., 2021), antioxidant capacity (Pérez et al., 2023), resistance to the necrophytic fungal pathogen *Alternaria dauci* (Tas, 2016), and several studies on seed germination under abiotic stress (Bolton et al., 2019; Bolton and Simon, 2019; Simon, 2019; Simon et al., 2021). In addition, the collection has been used for ecogeographic variation analysis (Mezghani et al., 2019), genomic core collection curation (Corak et al., 2019), and evaluation of genomic prediction strategies (Corak et al., 2023), as well as in studies of carrot CWR on subspecies identification (Spooner et al., 2014).

Genetic improvement of stand establishment in carrot is an achievable and desirable alternative strategy to weed mitigation and seed treatments, as these horticultural strategies have significant drawbacks with regards to expense, labor, and time for seed producers and growers. Evaluation of genetic resources is an essential activity that promotes their utilization and adaptation (Gepts, 2006). Germplasm repositories do not systematically evaluate agronomic traits in germplasm collections (Byrne et al., 2018) or morphological traits such as root color root and shape (Allender, 2019). Trait ontologies are essential to germplasm evaluation, as they provide consistent and shared vocabulary with agreed-upon definitions to accurately and consistently document and describe plant phenotypes (Shrestha et al., 2010; Walls et al., 2012). Previous trait ontologies for carrot, such as the now-defunct RoBuST, included traits such as carotene content, flavor, pungency, lutein, pathogen resistance, xylem/phloem color, and root shape (Bhasi et al., 2010). Above-ground vigor and biomass were not part of the traits included in that system. The recently established CarrotOmics database has a far more extensive suite of traits, including 280 traits defined, most of which pertain to the carrot root and very few characterize pertaining to the carrot shoot (Rolling et al., 2022). Given that recent studies found moderate heritability of shoot growth traits among cultivars (Luby et al., 2016) and mapping populations (Turner et al., 2018b), we hypothesized that heritable variation can be found in diverse carrot germplasm. Characterization of the germplasm collection broadens the genetic base that breeders can leverage in carrot breeding programs, and provides useful recommendations of accessions to include in breeding programs targeting improvement of seedling vigor, stand establishment, and weed competitive ability.

## Materials and methods

### Population under study

**Figure 1:**
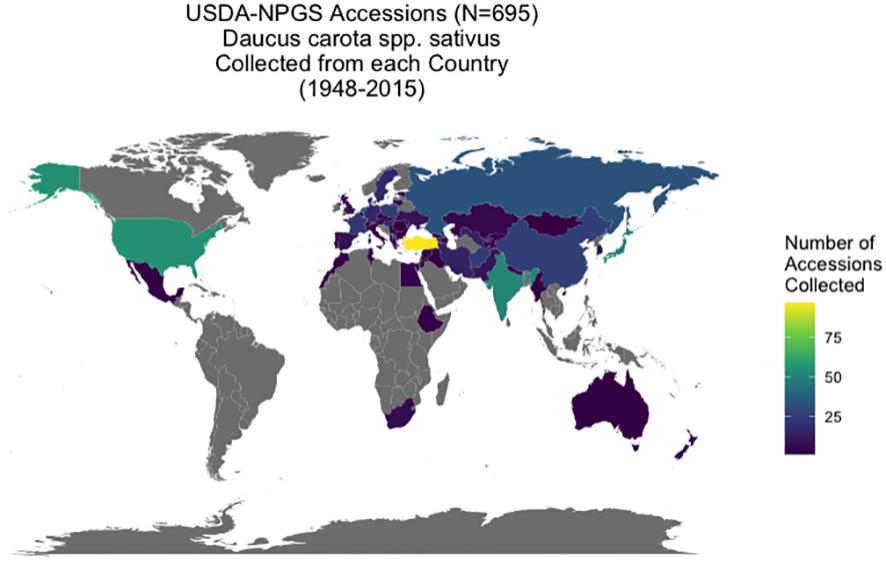
Origin of USDA-GRIN accessions (*Daucus carota* spp. *sativus*). Geographic distribution of *Daucus carota acc*essions collected from 60 countries between 1948 and 2015. These accessions have been cataloged in the GRIN-Global System and are maintained by the USDA North Central Regional Plant Introduction Station in Ames, IA.

*Daucus* accessions (N = 1381) are maintained through the USDA-NPGS at the North Central Regional Plant Introduction Station (NCRPIS) in Ames, IA, with information on the accessions in the Germplasm Resources Information Network (GRIN) database of the NPGS (GRIN-Global, 2023). From a genetic perspective, a carrot accession is a heterogeneous, heterozygous population increased by open-pollination and is genetically distinct from other accessions in the collection. This carrot collection represents global carrot germplasm, collected over multiple plant exploration trips between 1947 and 2015 from 60 countries. Over 80% of accessions originate from the Eurasian supercontinent: 53% from Asia, 34% from Europe and the Caucasus, and 13% (in descending order) were collected from the Americas, Africa, Australia, and New Zealand. At least 148 accessions originated in the primary center of diversity in central Asia (modern-day Afghanistan and surrounding countries) and secondary center of diversity in western Asia (modern-day Turkey) (Vavilov, 1951; Banga, 1963) (**Figure 1**). Passport data from GRIN-Global also provided seed viability (average germination percentage based on four independent replicates of 50 seeds per accession at standard germination conditions) and weight of 100 seeds in grams (average of two replicates of 100 seeds) for each accession (personal communication: Kathleen Reistma). Accessions were selected for this study’s diversity panel if they had passport information suggesting they were domesticated or exhibited domestication traits in preliminary screening, as evidenced by taproot traits such as increased pigmentation (carotenoid or anthocyanin), reduced lateral root branching, and increased taproot size (Ellison et al., 2018), resulting in 695 cultivated carrot accessions for this study. Many of these accessions are considered landraces or heirloom cultivars with annual, biennial, or mixed flowering habit. Although biennial flowering habit is a known domestication trait in carrot (Alessandro et al., 2013; Ellison, 2019), accession flowering habit data from the gene bank (annual, biennial, or mixed population) was of limited use due to having been phenotyped in various environments. Consequently, flowering habit was not a criterion used in curating this cultivated diversity panel. However, flowering habit data was collected during the course of this study and is the subject of this article’s companion paper (Loarca et al., 2024). A list of accessions used in this study from GRIN-Global is available (**Supplementary Table 6**) and raw data for each accession is available on the CarrotOmics database (Rolling et al., 2022).

### Experimental design

In mid-May in 2016, 2017, and 2018, we hand-planted one replicate of each accession in each of two blocks of a randomized complete block design (RCBD) at the Hancock Agricultural Research Station (ARS). Seeds were hand-planted in meter-length plots, in two adjacent hand-created furrows, 2 cm apart, at an approximate planting depth of 0.5 cm, with approximately 50 seeds per plot. Planting beds were prepared with a bed shaper. Each planting bed was 6 meters long and 1.7 meters wide, which provided enough space for 18 1-meter plots (6 plots lengthwise and 3 plots widthwise). Rye grass was planted in between the plot rows to maintain the structure of the sandy beds. Weeds were suppressed throughout the season with regular herbicide application. The field was watered with overhead irrigation. In 2016, plots with more than 50 plants were thinned to 50 plants per plot. This research station is in the central sands region of central Wisconsin, which is the third largest carrot producing state in the U.S. ($8.5M; 4,100 acres; 92K metric tons produced), making this a highly relevant target environment for identifying high-performing accessions (USDA Economic Research Service (ERS), 2023). This region has a characteristic sandy soil that is amenable to carrot seedling emergence and reflects optimal conditions for carrot cultivation.

### CarrotOmics shoot-growth ontology and trait evaluation

**Figure 2:**
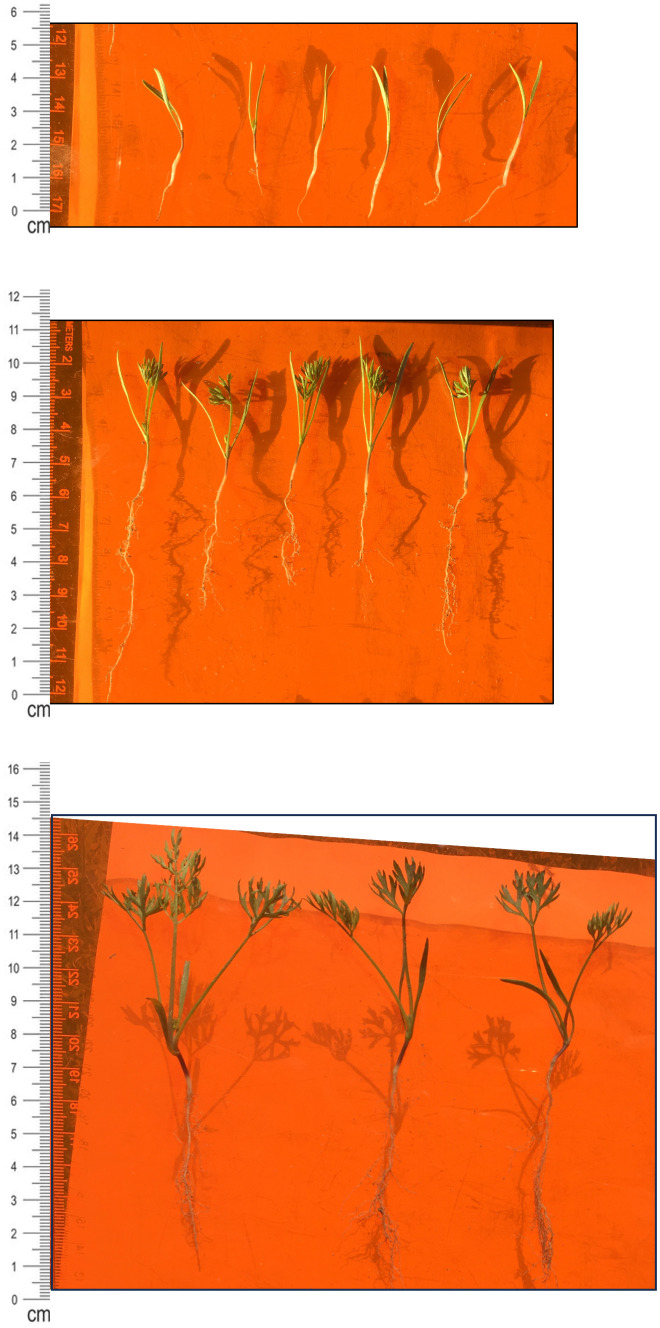
Carrot Seedling Development. (top) Week 1 seedlings (cv. Bolero). Coleoptile and mesocotyl approx. 2-2.5 cm in length. Radicle approx. 1-1.5 cm in length. First true leaf at the base of coleoptiles is barely visible to the naked eye. Scale marker on left in centimeters. Artifacts in the background are seedling shadows. (middle) Week 2 seedlings (cv. Bolero). Coleoptile and mesocotyl approx. 3.5-4 cm in length. Radicle approx. 3-6 cm in length. True leaves (1-2) are clearly visible. Scale marker on left in centimeters. (bottom) Week 3 seedlings (cv. Bolero). Coleoptile and mesocotyl approx. 6-8 cm in length. Radicle approx. 5-7 cm in length. True leaves (2-4) are clearly visible. Scale marker on left in centimeters.

**Figure 3:**
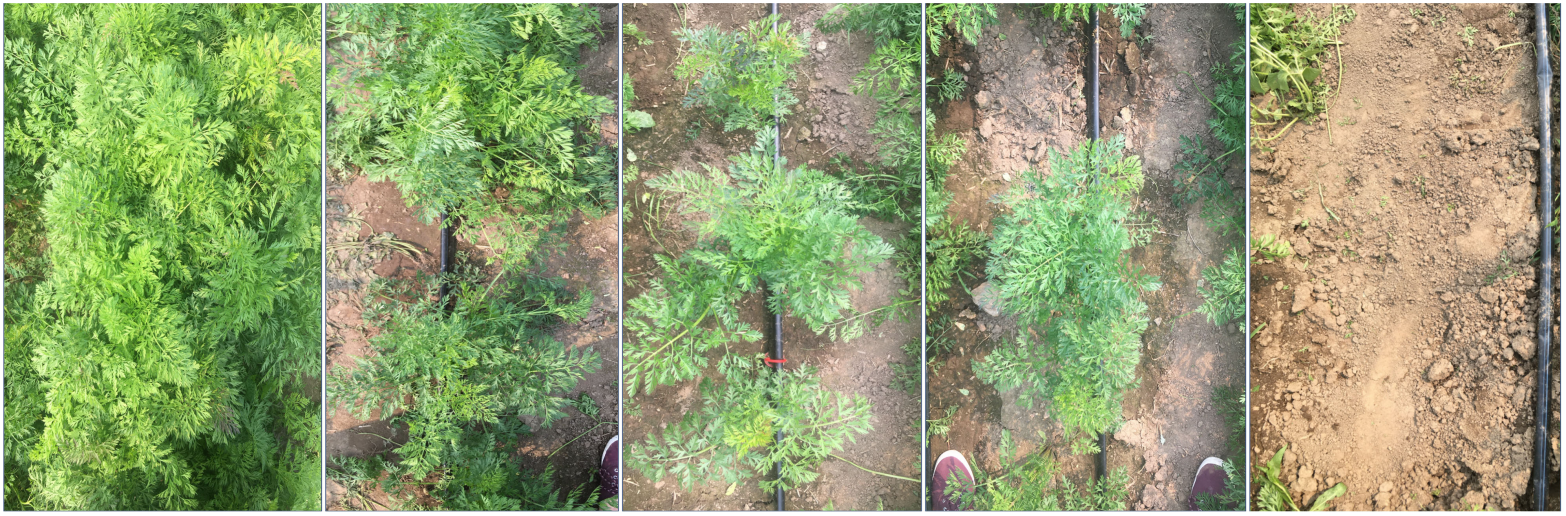
Canopy coverage is defined by the CarrotOmics shoot-growth ontology as the proportion of the ground covered by carrot foliar biomass. Photograph taken approx. 150 cm above the meter-length plot. Photos below are from five independent plots with canopy coverage in descending order from 100% carrot canopy coverage (left) to 0% carrot canopy coverage (right).

We included new shoot growth traits and descriptions, as well as elaborations to previously defined traits in the CarrotOmics database (Rolling et al., 2022). Traits measured (**Table 1**) include stand count (20 DAS), percent emergence (20 DAS), canopy height (40, 80, and 100 DAS), and canopy coverage (50, 80 and 100 DAS). In **Table 1**, these traits are described alongside existing trait descriptions in CarrotOmics. Stand count (20 DAS) refers to the total number of plants with cotyledons emerged in each plot for each accession. Emergence percentage is a transformation of stand count, calculated from the observed stand count divided by the expected number of plants (i.e., the number of seeds planted; approx. 50) (**Figure 2**). Canopy height (80 and 100 DAS) was measured on each plot at three randomly selected points within the plot, from root shoulder to top of leaf canopy (Turner et al., 2018a). We defined ‘canopy coverage’ as a visual estimate of the proportion of the soil obscured by carrot top-growth vegetation when viewed from a single point above the plot (**Figure 3**). We evaluated canopy coverage (50 DAS) using a five-point scoring system (0%, 25%, 50%, 75%, or 100%).

**Table 1:** CarrotOmics shoot-growth trait ontology developed in this paper, compared with current trait descriptions in GRIN-Global This paper elaborates on traits that were previously recognized as important in CarrotOmics and provides standard methodologies that carrot researchers can follow, enabling collaboration across programs.

CarrotOmics Trait Ontology for Shoot-Growth	2023 GRIN-Global Shoot-Growth Trait Descriptors	
**Stand Count** Absolute number of seedlings emerged within a field plot. 14 (early vigor), 20 (standard measurement)	**Seedling Vigor** “1=good” (GRIN-Global)	
**Percentage Emergence** Stand count divided by the number of seeds planted in plot 14 (early vigor), 20 (standard measurement)	**– –**	
**Canopy Coverage** Visual estimate of the proportion of the soil obscured by carrot top-growth vegetation when viewed from a single centered point above the field plot at notetaker’s eyeline. Measured visually on a 0% - 100% scale in increments of 25%. 50 (early-season), 80 (late-season), 100 (harvest day)	**Shoot Biomass** “Estimate of mass of all shoot tissue more than 4 cm above the crown, obtained from image analysis” (Turner 2018a).	
**Canopy Height** Measured at various times throughout the season, with three random measurements taken per plot. 40 (early vigor), 80 (late-season vigor), 100 (harvest day)	**Canopy Height at Harvest** “Canopy height was measured just before harvest with three measurements taken per plot” (Turner 2018a).	

Trait evaluation time is given in days after seeding (DAS) that the measurement was taken. Expected data collection times for the same plant growth stage in other programs may vary by location, cultivar, market type, and length of growing season.

### Data management

All statistical analyses were performed using RStudio Version 2023.6.1.524 (Posit team, 2023) and R Version 4.3.1 (R Core Team, 2023). Rosner’s Test in the *EnvStats* identified multiple simultaneous potential outliers for each trait in each year (Millard, 2013). Data was subset and manipulated with the *tidyverse* suite of packages (Wickham et al., 2019). Rosner’s Test implemented in *EnvStats* was used to identify multiple possible outliers for each trait in each year (Millard, 2013). A variety of utility packages were critical to data analysis, including *ggthemes* (Arnold, 2021), *beepr* (Bååth, 2018), and *flextable* (Gohel and Skintzos, 2023).

### Analysis of variance

F-tests of significance were performed using fixed effects models in a two-way Analysis of Variance (ANOVA) with Type III sums of squares using the car package (Fox and Weisberg, 2019). For each year, a fixed effects model was structured to calculate the proportion of phenotypic variance for each trait attributable to genotype: $T_{ik}=u+g_{i}+b_{k}+e_{ik}$ , where T = phenotypic measurement of the trait of interest (emergence, canopy coverage, or canopy height), g_i_ = genotype, b_k_ = block, and e_ik_ = error with e_ik_ ~ i.i.d. N(0, σ^2^).

The multi-year fixed effects model included trait data from multiple years and calculated the proportion of phenotypic variance in each trait attributable to *g_i_* = genotype, *y_j_* = year, *(gy)_ij_* = genotype*year interaction, *b_k(j)_* = block within year: $T_{ijk}=u+g_{i}+y_{j}+{\left(gy\right)}_{ij}+\;b_{k\left(j\right)}+e_{ijk}$ , where T= phenotypic measurement of the trait of interest and *e_ijk_* = error with *e_ijk_* ~ i.i.d. N(0, σ^2^). Due to unbalanced data from abnormal weather events (destructive hail), we ran two multi-year ANOVAs: one that included the 2017 shoot-growth data and one that excluded the 2017 shoot-growth data.

### Broad-sense heritability and variance components

Variance components were estimated for each trait within-year (single-year) and across-years (multi-year) using random effects models with the lme4 package (Bates et al., 2015). Variance components for each trait were then used to calculate broad-sense heritability (H^2^) within and across years. Statistical analysis used the same models as for the Analysis of Variance described in the previous section, but with all effects random. Broad-sense heritability (H^2^) for each trait, within years (single-year model) and across years (multi-year model), was calculated from variance components, including genotypic variance (V_g_) and phenotypic variance (V_p_). As in the fixed effects ANOVA, we ran two multi-year analyses: one that included the 2017 data and one that excluded the 2017 data.

Single-year broad-sense heritability was calculated for each trait:


$$H^{2}\;=\;\frac{V_{g}}{V_{p}}=\;\frac{V_{g}}{V_{g}+\;\frac{V_{error}}{\#\;rep}}$$

Multi-year broad-sense heritability was calculated for each trait:


$$H^{2}\;=\;\frac{V_{g}}{V_{p}}=\;\frac{V_{g}}{V_{g}+\;\frac{V_{gy}}{\#\;years}+\frac{V_{error}}{\#\;years\;*\;\#\;reps}}$$

### Mixed models and estimated marginal means

**Figure 4:**
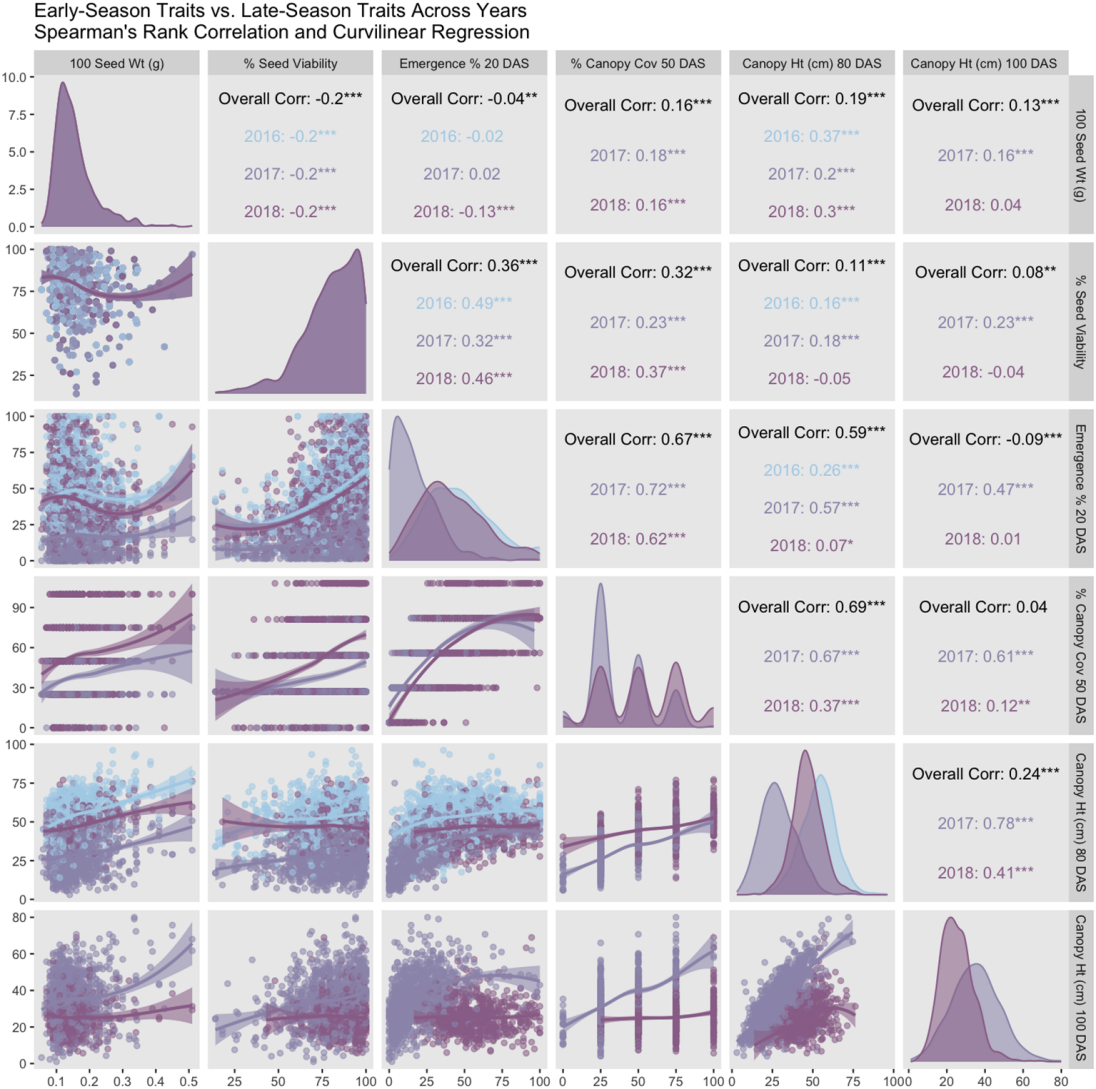
Pearson correlation matrix and curvilinear regression of early-season vs. late-season traits (2016-2018). Coefficients in the matrix indicate high correlation between both early-season traits. Similarly, both late-season traits are highly correlated. However, in normal years (2016 and 2018), both early-season traits are correlated weakly, if at all, with late-season traits. Anomalously, correlation is high between all traits in 2017, which is a year with poor stand establishment.

For each year and each trait, mixed models were fit using the same models as above with genotype as fixed effect and year and block as random effect (Bates et al., 2015). Estimated marginal means for each trait within each year were extracted from this model using the emmeans package (Lenth, 2023). Pearson’s sample correlation was use to calculate trait relationships and stability across years. Smoothed curves between traits were fit using Locally Estimated Scatterplot Smoothing (LOESS). Correlation coefficients (upper panels), curvilinear regression (lower panels), and trait distributions across years (diagonals) are summarized in a correlation matrix (**Figure 4**). These single-year estimated marginal means were used as phenotypes and summarized in **Table 2**. As in the prior models, we ran two multi-year mixed models: one that included the 2017 shoot-growth data and one that excluded the 2017 shoot-growth data.

**Table 2:** Descriptive statistics for early-mid season traits and late season traits.

CarrotOmics Shoot Growth Traits	2016		2017		2018		2016 & 2018	
	mean	SD	mean	SD	mean	SD	mean	SD
Emergence % (20 DAS)	46.38	21.82	17.23	14.98	41.73	21.69	44.67	16.63
Stand Count (20 DAS)	23.19	10.91	9.48	8.24	22.95	11.93	22.33	8.32
Canopy Coverage % (50 DAS)	-	-	37.55	20.61	52.47	26.73	-	-
Canopy Height (cm) (80 DAS)	53.42	10.98	28.76	10.84	46.62	9.15	49.47	9.49
Canopy Height (cm) (100 DAS)	-	-	35.08	12.65	25.46	8.73	-	-

Trait evaluation time is given in days after seeding (DAS) that the measurement was taken. Canopy coverage was measured on one replication in 2017. Late season canopy height was only recorded on accessions with stand count > 20 plants in 2018.

## Results

### Descriptive statistics

Germination data was collected by NCRPIS for accessions planted in this trial (79.7% ± 15.9%). Trait averages were consistent in 2016 and 2018 (42% - 46% emergence, 49 - 54 cm height, and 52% canopy coverage) (**Table 2**). Average emergence at 20 DAS (**Table 2**) is consistent with previous reports in carrot (35% - 77%) (Heydecker, 1956). Average canopy coverage (50 DAS) is within range of previous reports (32.5% - 80%) (Colquhoun et al., 2017), though we report a wider breadth of canopy coverage observations (0% - 100%). In 2017, average emergence (17%), canopy height at 80 and 100 DAS (28 cm and 35cm, respectively), and canopy coverage (37%) were extremely low compared to 2016 and/or 2018. Diagonal panels in **Figure 4** convey that trait distributions are visually consistent with descriptive statistics, demonstrating that trait performance (average and variance) are similar for 2016 and 2018, while 2017 observations are lower for all traits.

### Analysis of variance and broad-sense heritability

ANOVA results for all traits and years indicated that genotype was a highly significant factor (p< 0.001) influencing phenotypic variation. Broad-sense heritability estimates consistently indicated moderately high heritability across all years (**Table 3**) for emergence (0.61 < H^2^< 0.72) and canopy coverage (H^2^ = 0.65). Because emergence percentage is a transformation of stand count, their ANOVA outputs broad-sense heritability values were identical; we chose to present data only on emergence percentage. which is more generalizable to other studies and more intuitive than stand count. Broad-sense heritability estimate for canopy height was moderately high for all years at 80 DAS (0.64< H^2^< 0.82) (**Table 4A**) and at harvest day (100 DAS) (0.77< H^2^< 0.78) (**Table 4B**). These estimates are consistent with previous studies of end-of-season canopy height heritability estimates in carrot (0.65 < H^2^< 0.82) (Luby et al., 2016; Turner et al., 2018b). ANOVA results indicated that genotype is a highly significant source of canopy height variation, both in single-year (**Tables 4A**, **B**) and multi-year (**Table 5**) models. When excluding 2017 data, the year and block in year terms are not significant, however genotype and genotype x year effects remained highly significant (**Table 6**). Calculated p-values for shoot-growth ANOVA results are available in **Supplementary Tables 1**-**5**.

**Table 3:** ANOVA and broad-sense heritability (H^2^) for seedling emergence and canopy coverage.

Source of Variation	Emergence % (20 DAS)									Can. Cov. (50 DAS)		
	2016			2017			2018			2018		
	df	F	p	df	F	p	df	F	p	df	F	p
Genotype	684	3.50	***	693	2.59	***	693	2.70	***	694	2.80	***
Block	1	3.78	0.052	1	90.44	***	1	1.17	NS	1	10.39	***
Residuals	684			691			674			682		
**H^2^**	0.72			0.61			0.63			0.65		

ANOVA results indicate that genotype is a highly significant factor in all years. Broad-sense heritability (H^2^) is moderately high. P-values available in **Supplementary Table 1**.

**Table 4A:** ANOVA and broad-sense heritability (H^2^) of canopy height (80 DAS) (2016-2018).

Source of Variation	Canopy Height (cm) 80 DAS								
	2016			2017			2018		
	df	F	p	df	F	p	df	F	p
Genotype	648	5.46	***	674	2.72	***	458	3.24	***
Block	1	0.74	NS	1	106.54	***	1	0.14	NS
Residuals	616			591			270		
**H^2^**	0.82			0.64			0.74		

Statistically significant at * = p< 0.05; ** = p< 0.01; *** = p< 0.001; NS = otherwise.

ANOVA results indicate that genotype is a highly significant factor for late-season canopy height and broad-sense heritability (H^2^) is moderately high for all three years studied. P-values available in **Supplementary Table 2**.

**Table 4B:** ANOVA and broad-sense heritability (H^2^) of canopy height (100 DAS) (2017 & 2018).

Source of Variation	Canopy Height (cm) 100 DAS					
	2017			2018		
	df	F	p	df	F	p
Genotype	624	4.09	***	401	3.82	***
Block	1	5.95	*	1	13.04	***
Residuals	508			226		
**H^2^**	0.77			0.78		

Statistically significant at * = p< 0.05; ** = p< 0.01; *** = p< 0.001; NS = otherwise.

ANOVA results indicate that genotype is a highly significant factor for late-season canopy height and that broad-sense heritability (H^2^) is moderately high for all years studied, despite the presence of the disease, ALB. P-values available in **Supplementary Table 3**.

**Table 5:** Multi-year ANOVA and broad-sense heritability (H^2^) of emergence (20 DAS) and canopy height (80 DAS) (2016-2018), and canopy height (100 DAS) (2017 & 2018).

Source of Variation	Emergence (20 DAS)			Canopy Ht. (80 DAS)			Canopy Ht. (100 DAS)		
	df	F	p	df	F	p	df	F	p
Genotype	663	3.81	***	648	4.43	***	380	4.90	***
Year	2	9.53	***	2	15.76	***	1	0.22	NS
Genotype x Year	1328	1.63	***	1093	1.44	***	380	1.68	***
Block in Year	3	19.08	***	3	47.97	***	2	7.67	***
Residual	1974			1469			554		
**H^2^**	0.72			0.79			0.80		

Statistically significant at * = p< 0.05; ** = p< 0.01; *** = p< 0.001; NS = otherwise

ANOVA results indicated that genotype and genotype x year interaction were highly significant factors across all years. Broad-sense heritability (H^2^) is moderately high for all traits. P-values available in **Supplementary Table 4**.

**Table 6:** Multi-Year ANOVA and broad-sense heritability (H^2^) of emergence (20 DAS) and canopy height (80 DAS) (2016 & 2018).

Source of Variation	Emergence % (20 DAS)			Canopy Height (80 DAS)		
	df	F	p	df	F	p
Genotype	663	3.21	***	648	5.55	***
Year	1	1.26	NS	1	0.87	**NS**
Genotype x Year	662	1.54	***	439	1.30	***
Block in Year	2	1.65	NS	2	0.44	**NS**
Residual	1310			886		
**H^2^**	0.67			0.83		

Statistically significant at * = p< 0.05; ** = p< 0.01; *** = p< 0.001; NS = otherwise

ANOVA results indicated that, when excluding the abnormal 2017 data, year and block in year terms are not significant.-Genotype and genotype x year remained significant and broad-sense heritability (H^2^) is moderately high for all traits. P-values available in **Supplementary Table 5**.

### Trait correlations

Seedling emergence (20 DAS) and canopy coverage (50 DAS) demonstrated moderately strong correlation (0.62 < r < 0.72) across 2017 and 2018, despite poor emergence and poor stand in 2017. Seed viability (germination %) correlated moderately with seedling emergence (0.38 < r < 0.55).

In 2016 and 2018, emergence correlated poorly with canopy height 80 DAS (0.07 < r < 0.26) and canopy height 100 DAS (r < 0.013), while in 2017, correlations were moderate (0.47 < r < 0.58). Correlations with canopy coverage (50 DAS) were more variable with canopy height 80 DAS (0.37 < r < 0.67) and 100 DAS (0.12 < r < 0.62), with lower values representing normal years 2016 and 2018 and the higher value representing 2017 with poor stands overall. Curvilinear regression (**Figure 4**, lower panels) varies by year between emergence and canopy height, ranging from 0.07 < r < 0.57. Because 2017 had poor emergence, and 2018 did not record height on plots with fewer than 20 plants, it is likely that the 2016 dataset conveys the most accurate correlation (r = 0.256) between emergence and canopy height (80 DAS) in this study. Overall, early-season traits correlated highly with each other, and late-season traits correlated highly with each other (**Figure 4**, upper panels). Hundred-seed weight (g) did not correlate remarkably with any other traits in this study.

## Discussion

This study is the first multi-year study to systematically evaluate shoot-growth traits, from seed to harvest, in a global carrot diversity panel. This germplasm characterization has resulted in the identification of weed-competitive accessions that are of immediate utility to carrot breeders targeting improvement of stand establishment, particularly in organic carrots, and will result in reduced labor and cost associated with weeding, herbicide applications, and seed treatments. Strong broad-sense heritability for all traits measured indicates their potential to be improved through plant breeding. A program targeting early-season crop success would do well to focus on improving emergence. Strong correlation between emergence and canopy coverage suggests that improvement of seedling emergence has great potential to increase yield (through increased total number of individual carrot plants) and weed competitive ability (as all viable carrot plants contribute to canopy coverage). Accessions with vigorous emergence and canopy coverage provide breeders with raw materials for improving stand establishment in elite germplasm, increasing cultivar options for organic carrot farmers. Moderate correlation between germination percentage and seedling emergence suggests that, while necessary to produce a viable plant, it does necessarily predict successful emergence and crop establishment.

### Broad sense heritability

This study has demonstrated the breadth of variation for top growth traits present in a global carrot diversity panel, and is the first study to provide broad-sense heritability estimates for early-season seedling and shoot vigor. The broad sense heritability estimates we obtained demonstrate sufficient genetic control of emergence and vigor to be useful to breeding programs, however, this useful genetic variation may require breeders to use germplasm outside of current elite pools. We have also provided improved descriptions for agronomically-important shoot-growth traits in carrot, including standard methodologies and time-frames for trait evaluation. Data collection times were measured for central Wisconsin and will need to be adjusted according to location, cultivars, market type, and length of growing season. In addition, researchers will need to determine the optimal time-frames for trait evaluation for germplasm in their target locations. An inherent challenge of working with diverse germplasm of outcrossing crop species is intra-accession genetic variability as a source of unquantified variation in this study. Controlling for the level of inbreeding across accessions would correct the bias of inbreeding depression or hybrid vigor from recent outcrossing and high levels of accession heterozygosity.

Genotype was a highly significant factor in all traits in this study, demonstrating moderate to high broad-sense heritability for several agronomically important carrot shoot growth traits, and reporting the first broad-sense heritability estimates for seedling emergence (0.68< H^2^< 0.80) and canopy coverage (0.60< H^2^< 0.66). Heritability estimates for emergence fill the gap in heritability estimates identified by Dowker (1978). Our study contradicts Gray (1984), who claimed that genetic factors are not important when evaluating carrot seedling vigor on one carrot cultivar (cv. Red-cored Chantenay). Our results also disagree with a recent estimation that 0.6% of variation in emergence was explained by varietal identity, while 70% of variation was due to environment (Hundertmark-Bertaud et al., 2019). A major reason for their low estimate of genotypic variation could be due to their population under study, which included five F1 varieties of the market class Nantaise. Their conclusion that environmental conditions are more important than genetic background does not apply when evaluating a large genetically diverse and representative panel. All three years of our study indicated that genotype was a significant source of variation, and moderately-high broad-sense heritability for emergence supports our hypothesis that genetics have an important influence on variation in seedling vigor.

Broad-sense heritability for early-season canopy height (0.76 < H^2^ < 0.82) was similar to our broad-sense heritability estimate for late-season canopy height, and within range of plant height and canopy height estimates in previous studies (0.67 and 0.82, respectively) (Luby et al., 2016; Turner et al., 2018b). While these previous studies evaluated late-season carrot canopy height, our study provides the first broad-sense heritability estimates for early-season seedling vigor and early-season shoot vigor, which demonstrated high broad-sense heritability. Consequently, we recommend selection on early-season characteristics to improve weed competitiveness, with a goal of balancing rapid early season growth and moderate end of season biomass.

### Correlations among shoot growth traits

In 2017, a post-emergence hailstorm severely reduced stand, height, and canopy coverage. In 2016 and 2018, years in which hail damage did not occur, emergence correlated poorly with canopy height 80 DAS (0.07 < r < 0.26) and canopy height 100 DAS (r< 0.013), while in low-stand years like 2017, where hail damage occurred, correlations were more moderate (0.47 < r < 0.58) (**Figure 4**, upper panels). Years 2016 and 2018 were more normal years and comparable for stand count and late-season canopy height. However, the 2018 dataset excluded height on plots with fewer than 20 plants, and consequently the 2016 dataset likely conveys the most accurate correlation (r = 0.256) between emergence and canopy height (80 DAS) in this study. Emergence correlates weakly with late-season canopy height, which was unexpected, given the well-known density-dependent shade etiolation response, or shade avoidance, in plants.

Canopy coverage did not correlate with either canopy height measurements in years with normal weather. Low correlation between emergence and canopy height may also be explained by the nature of working with a diversity panel – there may be a variety of genetic responses to plant competitive conditions (Ballaré et al., 1994), including density-dependent self-thinning and shade tolerance (Westoby, 1984; Lonsdale, 1990). Similarly, canopy coverage has low correlation with both canopy height measurements. However, given that carrot tops have a unique morphology, with no internodes and long flexible petioles that bend under the weight of their own foliar growth (known as ‘canopy closure’), this may be unsurprising. This observation is consistent with Turner et al. (2018b), who also observed low correlation (0.3 < r < 0.4) between biomass and shoot height, as well as with shoot area and shoot height. Shoot biomass and shoot area, however, were highly correlated (r = 0.91).

Correlations between early-season traits and late-season traits were higher in 2017. This response is not consistent with low densities plots in 2016 or 2018. The 2017 results may accurately represent the kind of correlation that is typical of poor-stand years or when intentionally planted at low planting density. This response could indicate a tendency for these carrot shoots to grow into the space available, or to thrive in the absence of weed competition. Given that cultivated carrot competes poorly with weeds, this could be a reasonable and adaptive response to lack of competition. Further studies with multiple controlled densities would clarify this idea.

Previous studies measuring carrot canopy coverage reported an average canopy coverage of 66% and range of 32.5% - 80% at 55 DAS (Colquhoun et al., 2017). Our reported average canopy coverage (37% - 52%) was lower (which can be expected for unadapted germplasm) though we report a higher upper range (100%), which has important implications for improving canopy coverage through breeding. The greater variation reported for canopy coverage and emergence indicates that there are accessions in this collection with greater emergence and canopy coverage potential than some commercially available cultivars. The high correlation between emergence and canopy coverage suggests that seedling vigor may be an early-season predictor of mid-season canopy cover, crop vigor, and crop competitiveness.

### Factors interacting with measurement of shoot-growth traits

While height across the season may confer weed-competitiveness, it is not sufficient to select only for populations with the largest plants because excessive foliar biomass can impede the inner workings of machine harvesters. Furthermore, the advantage of early-season vigor can become a liability by late-season, as large canopies create a humid microclimate in which fungal pathogens, such as *Alternaria dauci*, a necrotrophic fungus that can readily infect leaves, causing Alternaria Leaf Blight (ALB) (Prohens and Nuez, 2008). ALB was present during all three years of our study, and is the most economically devastating carrot pathogen that is present in most carrot production areas (Tas, 2016). Therefore whole-plot mortality or foliage reduction caused by ALB had a confounding effect in late-season carrot shoot trait evaluation. Despite ALB’s destructive impact on foliar biomass, broad-sense heritability estimate for canopy height was still moderately high for all years at 80 DAS (0.64< H^2^< 0.82) (**Table 4A**) and at harvest (0.77< H^2^< 0.78) (**Table 4B**). It is not yet clear how these accessions would perform in an environment where this pathogen is well-controlled. Mitigating ALB as a source of noise would strengthen genetic signal for late-season canopy growth in future evaluations.

Despite high germination rates in this collection (79.7% ± 15.9%), average field emergence ranged from 42% - 52%, even in the presence of sufficient moisture​ and amenable soil and bed conditions. The gap between potential emergence and actual emergence has long been known in carrot (Heydecker, 1956; Hegarty, 1971; Finch-Savage and Pill, 1990; Finch-Savage et al., 1998), and the values reported in our study are consistent with previous studies (35% - 77% emergence) on untreated carrot seeds (Heydecker, 1956). We observed accessions with germination rates upward of 90% in replicated lab tests that demonstrated very low emergence and canopy coverage in the field. There are many potential mechanisms and points of failure between germination and emergence, that warrant significant attention in future studies (Steiner and Zuffo, 2019) in diverse carrot germplasm. Accessions or seed lots that have high germination under standardized or controlled laboratory conditions will not necessarily germinate or emerge under field conditions. Therefore, while high seed germination is a prerequisite to seedling growth, it does not necessarily predict successful seedling growth, development, and emergence in the field. Therefore, while studies on seed priming may improve carrot seed germination in controlled test conditions (Aazami and Zahedi, 2018; Sowmeya et al., 2018; Mahmood-ur-Rehman et al., 2020; Guragain et al., 2021; Muhie et al., 2021; Muhie et al., 2024), which is an important part of the puzzle, these studies will have stronger potential to identify vigorous, agronomically useful germplasm when combined with a field emergence study, to develop a complete package for early-season agronomic performance under real world conditions ​ (Simon et al., 2021). Similarly, recent germination studies could be improved upon (Aazami and Zahedi, 2018; Bolton and Simon, 2019; Bolton et al., 2019; Nijabat et al., 2023) by screening germplasm in field emergence trials, especially given the long-known and well-established gap between lab germination and field emergence (Heydecker, 1956; Hegarty, 1971; Finch-Savage and Pill, 1990; Finch-Savage and McQuistan, 1988). Additional traits to measure include seedling vigor and emergence speed (Acosta-Motos et al., 2021).

Breeders have traditionally relied on well-adapted germplasm for development of improved cultivars. Our study empowers breeders to identify accessions with desirable top-growth traits that can be leveraged to invigorate breeding or pre-breeding programs with useful genetic diversity for shoot-growth and weed-competitive traits. We recommend that breeders interested in improving season-long weed competitiveness incorporate these trait measurements in their breeding and variety trial evaluations. Additional metrics of emergence that incorporate growth uniformity have important implications for end-of-season root yield. Future studies on seedling vigor would benefit from additional measurements of uniformity, such as emergence timing and seedling growth rate. While studying emergence requires uniform seeding rate, studying canopy height requires uniform emergence to achieve uniform planting density, and consequently, uniform intraplant competitive conditions. This would necessitate overplanting accessions with low germination or low emergence to achieve uniform planting density conditions across all accessions.

Furthermore, optimal planting density varies depending on carrot root shape. While all accessions in this study had the same number of seeds per plot, future studies may benefit from considering the relationship between market class and planting density. This carrot germplasm collection was recently evaluated for root shape and market class, though not all cultivars fit cleanly into a particular market class (Brainard et al., 2021b). Bulkier carrots are grown at lower population densities than slimmer fresh market types at higher densities (1,500,000 - 3,000,000 plants per hectare) – these densities were established to produce high levels of biomass on a *per hectare* and a *per root* basis (Goldman, 2019). However, planting densities above the optimal rate can reduce individual root biomass by 50% (Vega and Goldman, 2023). Integrating this data with known optimal planting densities for carrot market classes in this germplasm collection, to the extent possible, would enable optimal plant density, rather than uniform seed rate – this is important because it is not yet known how planting density of various market types affects shoot growth. Accounting for planting density would enable accessions with similar optimal planting densities to be compared, given that planting density is well-documented as a source of variation in above-ground biomass and morphology (Duthie et al., 1999; Peil and López-Gálvez, 2002; Aziz et al., 2007; Goss, 2012; Khan et al., 2017; Postma et al., 2021). Non-optimal planting densities for the certain root shapes in this collection could partially explain the unexpected lack of correlation between stand count and height in our study.

### Measurement accuracy and labor

Stand count provides very valuable information about genotype performance because early emergence correlates with root uniformity at harvest (Mann and MacGillivray, 1949), one of the primary components of marketable root yield. The moderately high broad-sense heritability estimates reported in our study suggest that the current phenotyping methods we presented are capable of detecting a genetic signal among a diverse set of germplasm. However, stand count is the most physically laborious and time-consuming trait to measure in this study. The time required to phenotype one plot increases with emergence and planting density. Because carrot seedlings at 20 DAS are still very small (2-7 cm) and typically densely planted, stand count data collection requires technicians to bend, kneel, or squat over the plot. Moreover, counting requires manually separating the plants, which is disruptive to established seedlings.

Canopy coverage phenotyping is the fastest of all methods described, requiring fewer than five seconds to assign a value to each plot and can be recorded from an upright position (i.e., no bending or squatting required). Canopy height data collection requires less than one minute per plot with one technician, but is more efficient with two technicians, as one records data while the other reports the data. Moreover, because canopy height measurements can also require bending or squatting, sharing the load between two technicians reduces laborious repetitive motions. Other carrot studies have used a similar scoring method to visually estimate canopy coverage on a continuous scale (0% - 100%) to measure ‘ground cover’ or ‘carrot canopy development’ in an experiment that included nine entries (Colquhoun et al., 2017). In contrast to a continuous scale, the five-point visual scale improved phenotyping efficiency, which was critical on an experiment of this size. It is not clear how much more accuracy is gained from a continuous vs. categorical visual estimation. Evaluation of canopy coverage on a continuous scale could smooth the distribution for canopy coverage (**Figure 4**, diagonal panel for canopy coverage). Drone imaging could convert this measurement to a continuous trait, potentially improving estimates of canopy coverage, but requires significant investment in training, equipment, software, and analysis that not all programs necessarily have access to.

The canopy-coverage phenotyping method presented in this study demonstrated sufficiently high broad-sense heritability for canopy coverage (H^2 =^ 0.65) to begin to make progress on evaluations, selections, and genetic gain on canopy coverage. Beyond potential gains in phenotyping accuracy, image-based phenotyping of field plots would eliminate the risk of repetitive motion injuries while collecting data in the field. Unpiloted aerial vehicles (UAV) with a RGB camera would provide a high-throughput phenotyping method to evaluate shoot growth traits in carrot field trials. This method would increase measurement speed and provide a three-dimensional rendering of other shoot architecture traits, such as canopy height and canopy coverage, using common surface-from-motion algorithms. It is not yet known at what planting density high-quality cameras can resolve among individual carrot seedlings to accurately measure stand count or distinguish carrot seedlings from weeds. In our study, the few weeds in our field were visually ignored when making canopy coverage estimates. Digital phenotyping methods are under development to distinguish weeds from carrot shoots (Miao et al., 2019; Ying et al., 2021).

The CarrotOmics shoot-growth ontology definition of canopy coverage (**Table 1**) in the field is similar to Turner’s description of postharvest shoot biomass (Turner et al., 2018a). However, our definition of canopy coverage is not intended to supplant Turner’s methodology – both methods are appropriate for evaluating shoot biomass in different context, with the present method providing visual estimates of whole-plot shoot tissue biomass evaluated in the field, and Turner’s method estimating shoot biomass from images obtained in the lab after harvesting roots from the field. Both estimate carrot shoot biomass using two-dimensional data, and Turner’s method is suitable for postharvest evaluation in the lab, which can be photographed in standard lighting conditions and analyzed with imaging software (Turner et al., 2018b). The method described in the present study is suitable for season-long canopy estimation in the field.

## Conclusions and recommendations

We encourage carrot researchers to utilize and expand upon the descriptive terminology that we have developed. More shoot growth traits can be added to CarrotOmics shoot-growth ontology and more detailed aspects of crop growth and seedling morphology have been described and evaluated. Some traits can be studied as properties of emergence, such as emergence speed and emergence uniformity (Egli et al., 2010; Samfield et al., 1991; TeKrony and Egli, 1991). Additionally, studies in rice, wheat, and castor bean have evaluated seedling length, seedling weight, and growth rate (Hughes and Mitchel, 1987; Zhou et al., 2010; Abe et al., 2012; Guo et al., 2019). In wheat, coleoptile length has been implicated in seedling vigor (Rebetzke et al., 2007; Li et al., 2014), while in rice and pearl millet, mesocotyl elongation has been implicated in seedling vigor – an important aspect of successful stand establishment (Mohamed et al., 1989; Lee et al., 2012; Ohno et al., 2018). Genetic studies in rice and wheat have found quantitative trait loci (QTL) associated with seedling vigor (Zhang et al., 2005; Spielmeyer et al., 2007; Zhou et al., 2007; Landjeva et al., 2010).

Public availability of multi-year or multi-environment phenotypic data facilitates selection of accessions with desirable agronomic traits and can be used by researchers to create core collections (Berger et al., 2013; Byrne et al., 2018). The wealth of data available on this carrot germplasm collection enables identification of germplasm across a suite of agronomically important traits, such as flowering habit (Loarca et al., 2024), ALB resistance (Tas, 2016), beta-carotenes (Ellison et al., 2018), plant growth traits (Acosta-Motos et al., 2021), taproot shape (Brainard et al., 2021), antioxidant capacity (Pérez et al., 2023), and seed germination under abiotic stress (Simon et al., 2021). While results are specific to central Wisconsin, ranked correlations were high for two out of three years of our studies. It is unknown how these ranks would shift when evaluating this germplasm in other significant carrot growing regions globally, such as in other temperate growing regions, in subtropical climates, or in the other significant growing regions in the U.S. (Washington and California). We highly recommend application of our methodology to evaluate other global carrot germplasm collections and identify ecoregional adaptation for shoot growth vigor in each target environment.

This cultivated diversity panel was curated from a *Daucus* collection to increase the relevance of our germplasm evaluation to commercial breeders. In addition to variation for shoot growth phenotypes, this collection contains annual, biennial, and mixed flowering habits and has now been characterized with the CarrotOmics flowering habit trait ontology in the companion to this article (Loarca et al., 2024), in which relationships between carrot shoot vigor and flowering habit have also been elucidated. We have found locally adapted accessions with consistent performance over multiple years to start breeding pools for stand establishment, thereby lowering the barrier to utilization of carrot genetic resources. This list of accessions can be further optimized for efficient breeding in combination with phenological data using methods from this study’s companion paper to identify ideotypes based on global market needs, such as biennial accessions for temperate breeding programs or late-flowering annual accessions for semi-arid or subtropical breeding programs. Carrot global per capita production has increased 2.7-fold in the last fifty years (Simon, 2019), making this question of multi-environment trialing of diverse germplasm relevant to all global regions of carrot production.

## Data availability statement

A list of accessions used in this study is provided in **Supplementary Table 6**. Researchers may request these accessions through the USDA Germplasm Resources Information Network (GRIN) database of the U.S. National Plant Germplasm System (NPGS). https://npgsweb.ars-grin.gov/gringlobal/search. The datasets for this study are hosted on the CarrotOmics database https://www.carrotomics.org/file/409952.

## Author contributions

JL: Conceptualization, Data curation, Formal analysis, Investigation, Methodology, Project administration, Software, Supervision, Validation, Visualization, Writing – original draft, Writing – review & editing. ML: Formal analysis, Software, Validation, Writing – review & editing. JD: Conceptualization, Data curation, Formal analysis, Funding acquisition, Methodology, Project administration, Resources, Software, Supervision, Validation, Writing – review & editing. PS: Conceptualization, Funding acquisition, Methodology, Project administration, Resources, Supervision, Validation, Writing – review & editing.

## Supplementary material

Supplementary Table 1 P-values for ANOVA of seedling emergence and canopy coverage. ANOVA results indicate that genotype is a highly significant factor in all years. Broad-sense heritability (H^2^) is moderately high for early-season vigor in all years studied. Statistically significant p-values in bold.

Supplementary Table 2 P-values for ANOVA of canopy Height (80 DAS) (2016-2018). ANOVA results indicate that genotype is a highly significant factor for late-season canopy height in all three years studied. Statistically significant p-values in bold.

Supplementary Table 3 P-values for ANOVA of canopy height (100 DAS) (2017 & 2018). ANOVA results indicate that genotype is a highly significant factor for late-season canopy height despite the presence of the disease ALB. Statistically significant p-values in bold.

Supplementary Table 4 P-values for multi-year ANOVA for emergence and canopy height (80 DAS) (2016-2018) and canopy height (100 DAS) (2017 & 2018). ANOVA results indicated that genotype and genotype x year interaction were highly significant factors across all years. Statistically significant p-values in bold.

Supplementary Table 5 P-values for multi-year ANOVA of emergence (20 DAS) and canopy height (80 DAS) (2016 & 2018). ANOVA results indicated that the year and block in year terms became non-significant when excluding 2017 data from the model. Genotype and genotype x year interaction were statistically significant factors across all years. Statistically significant p-values in bold.

Supplementary Table 6 List of Plant Introductions (also known as accessions or genotypes) (N = 695) used in this study.
